# The structural science of functional materials

**DOI:** 10.1107/S2052252517018474

**Published:** 2018-01-01

**Authors:** C. Richard A. Catlow

**Affiliations:** a Department of Chemistry, University College London, 20 Gordon Street, London WC1H 0AJ, UK; b School of Chemistry, Cardiff University, Cardiff, CF10 3AT, UK

**Keywords:** functional materials, structural science, editorial

## Abstract

The growing complexity of functional materials and the major challenges this poses to structural science are discussed. The diversity of structural materials science and the contributions that computation is making to the field are highlighted.

The growing complexity of many classes of functional materials poses major challenges to structural science to which technical developments and the increasingly predictive power of computation make key contributions. These themes are clearly illustrated by the articles published in the field during the last year and especially by those in **IUCrJ**.

An excellent illustration of the complexity of structural problem that is now being investigated in materials science is provided by the work of Zhang, Zhang *et al.* (2017 ▸), who examine the structural features of the martensitic transformation in Ni/Mn/Sb intermetallic compounds. The properties of such alloys are modified and can be optimized by the transformation, which alters the microstructure. The study uses scanning electron microscopy/electron back diffraction to chart the fascinating structural organizations that are induced, providing detailed structural models of these complex and intriguing transitions. The theme of phase transitions in intermetallics is also taken up in the paper of Li *et al.* (2018 ▸), who examine Mn/Co/Ni/Sn melt-spun ribbons and link phase behaviour to magneto-caloric properties; while the impact in this field of computation is apparent from the study of Wang *et al.* (2017 ▸), who report detailed electronic structure studies of a series of Heusler compounds, discussing the tuning of band structure for spintronic applications. Structure–property relations in this class of compound are further explored in the study of Qin *et al.* (2017 ▸) on the effects of ‘swap disorder’ on the physical properties of Pd/Mn/Ti/Al Heusler alloys. The interweaving of advanced techniques with predictive computation is very evident in the growing body of work on intermetallics.

A field in which complexity has been long recognized is that of perovskite-structured materials, especially relating to the rich phase behaviour of compounds based on this versatile structural type. The lead zirconate/titanate solid solution (PZT) has been intensively studied over several decades, owing both to the subtlety of its structural properties and to the very wide ranging applications arising from its piezoelectric behaviour. New insight into structural changes and phase relations is provided by the study of Zhang, Yokota *et al.* (2017 ▸). Using a pair-distribution-function analysis based on neutron data, this paper shows how the local structure in PZT controls the long-range average structure across the so-called morphotropic phase boundary; and the analysis discovers a new monoclinic *M_C_* type structure. In addition, the role of polarization rotation in increasing the piezoelectric properties is analysed. Fig. 1 ▸ gives a diagrammatic illustration of the Pb polarization rotation paths that are revealed by the study.

The paper represents a particularly elegant example of the elucidation of structure–property relations in current materials science; while the continuing challenges posed by perovskite-structured materials is also illustrated in the work of Kang *et al.* (2017 ▸), which discovers new and unexpected spin configurations in magnetic phase transitions in Mn-doped SmFeO_3_.

The theme of structural changes during phase transitions again forms the basis of the work of Matvienko *et al.* (2017 ▸), which returns to structural changes during martensitic transitions and examines the role that optical microscopy can play in elucidating the mechanisms of transition by monitoring the interface migration and change in crystal shape during a transition. The approach is illustrated by the changes taking place during the dehydration of hydrated samarium oxalate. Fig. 2 ▸ shows some of the information and insight obtained from this study.

The characterization of disorder remains a perennial challenge in the structural science of materials. The work of Kim *et al.* (2018 ▸) provides a very significant contribution to this field. They report on atomic resolution strain analysis for vacancy detection in InAs/GaSb strained super-lattices with high-angle annular-dark-field (HAADF) imaging in a scanning transmission electron microscope (STEM). The ability to map strain fields around vacancies as demonstrated in this paper will have implications for other systems and also represents a challenge to theory and modelling.

The determination of structure–function relationships in nano structures is an active and growing field, especially in supported nano-structured catalytic materials. Here, in addition to TEM, X-ray spectroscopy is providing powerful tools as illustrated by the recent work of Dann *et al.* (2017 ▸) and Rogers *et al.* (2017 ▸), which addresses the structures and structural variations during synthesis of palladium and palladium oxide nano particles used in catalytic applications. The latter study shows how the solvent employed in the colloidal synthesis of the supported nano structures can influence particle morphology and catalytic applications. Structure–function relationships are also crucial in the large and growing field of materials for applications in energy technologies, as is well illustrated in the review of Peterson *et al.* (2017 ▸), which highlights the ability to undertake real-time diffraction studies of both battery electrodes and porous sorbent materials.

Computation now pervades all aspects of structural science. The growing power of structure prediction methods is highlighted by the recent work of Collins *et al.* (2017 ▸), which uses techniques embracing data mining and electronic structure methods that are employed together with powder diffraction techniques to identify the structures of two new phases in the complex Y–Sr–Ca–Ga–oxide phase field.

Bond-valence methods continue to provide a simple but useful approach to understanding and validating inorganic crystal structures. Chen & Adams (2017 ▸) report a new detailed parameter set for the approach, which is discussed further by Brown (2017 ▸), who urges further work to understand the nature of the bond-length–bond-valence correlation.

Further illustration of the role of current computational methodologies is provided by the study of Ma *et al.* (2017 ▸), which uses electronic structure techniques to understand the relationship between molecular packing and charge transport parameters in organic molecular crystals – a topic of importance in solar-cell and light-emitting-diode materials.

It is of interest to note how machine-learning techniques are contributing to the field as in the work of Park *et al.* (2017 ▸), which uses a neural network in the classification of X-ray diffraction data in terms of crystal system, extinction group and space group. The trained neural net can then be used to derive symmetries of new inorganic compounds. Applications of machine-learning techniques in the field will unquestionably grow.

In conclusion, it is hoped that this brief editorial highlights the diversity of structural materials science and the contributions which computation is making to the field. Articles based on these topics will continue to be welcomed by **IUCrJ**.

## Figures and Tables

**Figure 1:**
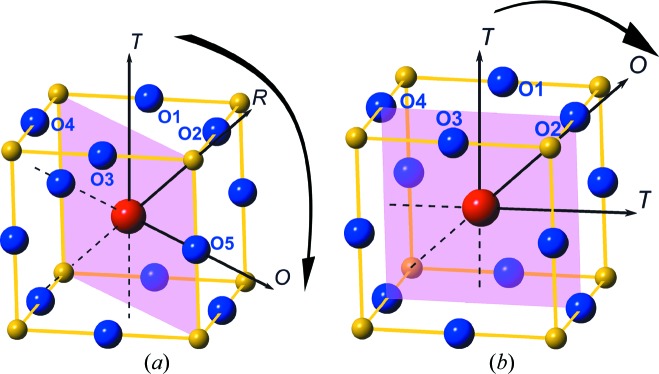
Schematic diagrams of the polarization rotation paths for Pb displacement vectors in the monoclinic mirror planes in the (*a*) *M_A_*/*M_B_* and (*b*) *M_C_* structures discussed in Zhang, Yokota *et al.* (2017 ▸). The large red atom at the centre of the unit cell is Pb, while the blue atoms are the surrounding O atoms of the PbO_12_ cage. The small yellow atoms are the *B*-site cations Zr or Ti. Arrows indicate possible vector directions.

**Figure 2:**
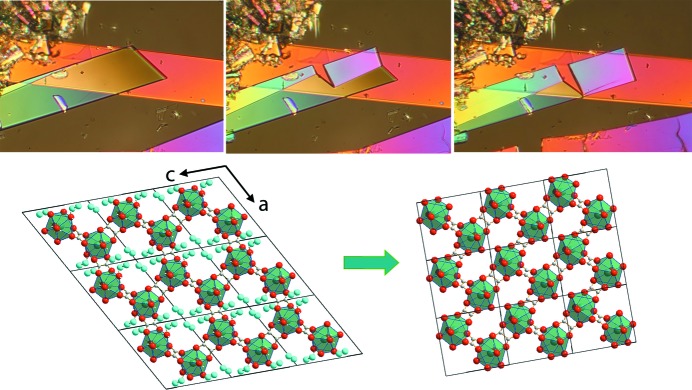
(Top) Optical micrographs of Sm_2_(C_2_O_4_)_3_·10H_2_O during dehydration to Sm_2_(C_2_O_4_)_3_·6H_2_O on heating. (Bottom) The orientation of the fragments of the crystal structures corresponds to the crystal shape. The hexahydrate crystal remains in the same plane. Figure taken from Matvienko *et al.* (2017 ▸).
